# Supplementary figures and images for: AMCFCN: attentive multi-view contrastive fusion clustering net (part 2 of 3)
Source: PeerJ Comput Sci. 2024 Mar 5;10:e1906. doi: 10.7717/peerj-cs.1906 (PMC11636696; doi:10.7717/peerj-cs.1906)

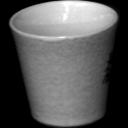

Supplement: Supplemental Information 1 [file peerj-cs-10-1906-s001.zip › coil-20/obj12__49.png]

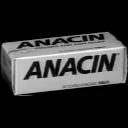

Supplement: Supplemental Information 1 [file peerj-cs-10-1906-s001.zip › coil-20/obj5__68.png]

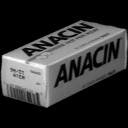

Supplement: Supplemental Information 1 [file peerj-cs-10-1906-s001.zip › coil-20/obj5__61.png]

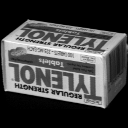

Supplement: Supplemental Information 1 [file peerj-cs-10-1906-s001.zip › coil-20/obj9__40.png]

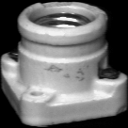

Supplement: Supplemental Information 1 [file peerj-cs-10-1906-s001.zip › coil-20/obj14__32.png]

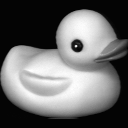

Supplement: Supplemental Information 1 [file peerj-cs-10-1906-s001.zip › coil-20/obj1__42.png]

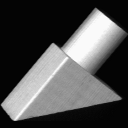

Supplement: Supplemental Information 1 [file peerj-cs-10-1906-s001.zip › coil-20/obj2__31.png]

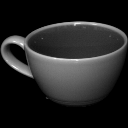

Supplement: Supplemental Information 1 [file peerj-cs-10-1906-s001.zip › coil-20/obj18__40.png]

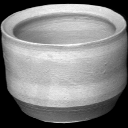

Supplement: Supplemental Information 1 [file peerj-cs-10-1906-s001.zip › coil-20/obj17__44.png]

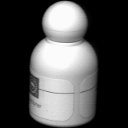

Supplement: Supplemental Information 1 [file peerj-cs-10-1906-s001.zip › coil-20/obj16__12.png]

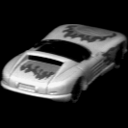

Supplement: Supplemental Information 1 [file peerj-cs-10-1906-s001.zip › coil-20/obj6__23.png]

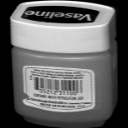

Supplement: Supplemental Information 1 [file peerj-cs-10-1906-s001.zip › coil-20/obj10__38.png]

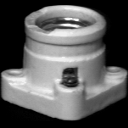

Supplement: Supplemental Information 1 [file peerj-cs-10-1906-s001.zip › coil-20/obj14__7.png]

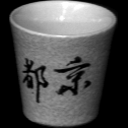

Supplement: Supplemental Information 1 [file peerj-cs-10-1906-s001.zip › coil-20/obj12__0.png]

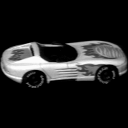

Supplement: Supplemental Information 1 [file peerj-cs-10-1906-s001.zip › coil-20/obj6__34.png]

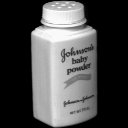

Supplement: Supplemental Information 1 [file peerj-cs-10-1906-s001.zip › coil-20/obj8__64.png]

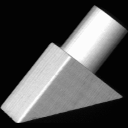

Supplement: Supplemental Information 1 [file peerj-cs-10-1906-s001.zip › coil-20/obj2__30.png]

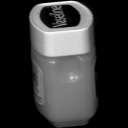

Supplement: Supplemental Information 1 [file peerj-cs-10-1906-s001.zip › coil-20/obj10__52.png]

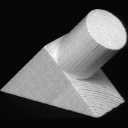

Supplement: Supplemental Information 1 [file peerj-cs-10-1906-s001.zip › coil-20/obj2__43.png]

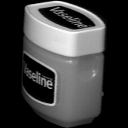

Supplement: Supplemental Information 1 [file peerj-cs-10-1906-s001.zip › coil-20/obj10__11.png]

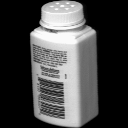

Supplement: Supplemental Information 1 [file peerj-cs-10-1906-s001.zip › coil-20/obj8__42.png]

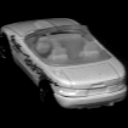

Supplement: Supplemental Information 1 [file peerj-cs-10-1906-s001.zip › coil-20/obj19__13.png]

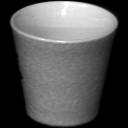

Supplement: Supplemental Information 1 [file peerj-cs-10-1906-s001.zip › coil-20/obj12__21.png]

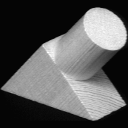

Supplement: Supplemental Information 1 [file peerj-cs-10-1906-s001.zip › coil-20/obj2__44.png]

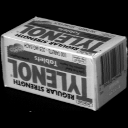

Supplement: Supplemental Information 1 [file peerj-cs-10-1906-s001.zip › coil-20/obj9__42.png]

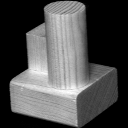

Supplement: Supplemental Information 1 [file peerj-cs-10-1906-s001.zip › coil-20/obj7__42.png]

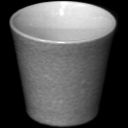

Supplement: Supplemental Information 1 [file peerj-cs-10-1906-s001.zip › coil-20/obj12__25.png]

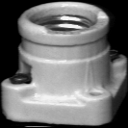

Supplement: Supplemental Information 1 [file peerj-cs-10-1906-s001.zip › coil-20/obj14__56.png]

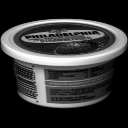

Supplement: Supplemental Information 1 [file peerj-cs-10-1906-s001.zip › coil-20/obj20__69.png]

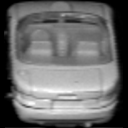

Supplement: Supplemental Information 1 [file peerj-cs-10-1906-s001.zip › coil-20/obj19__17.png]

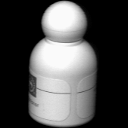

Supplement: Supplemental Information 1 [file peerj-cs-10-1906-s001.zip › coil-20/obj16__13.png]

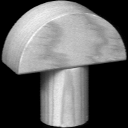

Supplement: Supplemental Information 1 [file peerj-cs-10-1906-s001.zip › coil-20/obj11__67.png]

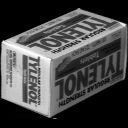

Supplement: Supplemental Information 1 [file peerj-cs-10-1906-s001.zip › coil-20/obj9__25.png]

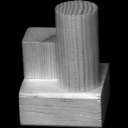

Supplement: Supplemental Information 1 [file peerj-cs-10-1906-s001.zip › coil-20/obj7__37.png]

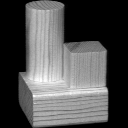

Supplement: Supplemental Information 1 [file peerj-cs-10-1906-s001.zip › coil-20/obj7__69.png]

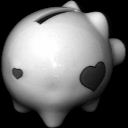

Supplement: Supplemental Information 1 [file peerj-cs-10-1906-s001.zip › coil-20/obj13__27.png]

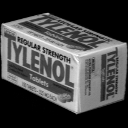

Supplement: Supplemental Information 1 [file peerj-cs-10-1906-s001.zip › coil-20/obj9__9.png]

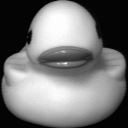

Supplement: Supplemental Information 1 [file peerj-cs-10-1906-s001.zip › coil-20/obj1__53.png]

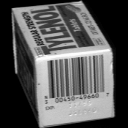

Supplement: Supplemental Information 1 [file peerj-cs-10-1906-s001.zip › coil-20/obj9__51.png]

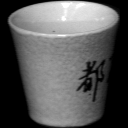

Supplement: Supplemental Information 1 [file peerj-cs-10-1906-s001.zip › coil-20/obj12__57.png]

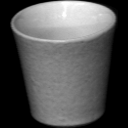

Supplement: Supplemental Information 1 [file peerj-cs-10-1906-s001.zip › coil-20/obj12__40.png]

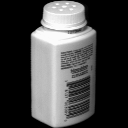

Supplement: Supplemental Information 1 [file peerj-cs-10-1906-s001.zip › coil-20/obj8__27.png]

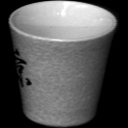

Supplement: Supplemental Information 1 [file peerj-cs-10-1906-s001.zip › coil-20/obj12__16.png]

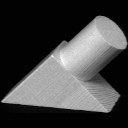

Supplement: Supplemental Information 1 [file peerj-cs-10-1906-s001.zip › coil-20/obj2__39.png]

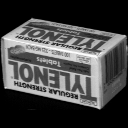

Supplement: Supplemental Information 1 [file peerj-cs-10-1906-s001.zip › coil-20/obj9__41.png]

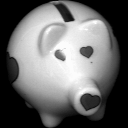

Supplement: Supplemental Information 1 [file peerj-cs-10-1906-s001.zip › coil-20/obj13__50.png]

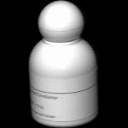

Supplement: Supplemental Information 1 [file peerj-cs-10-1906-s001.zip › coil-20/obj16__48.png]

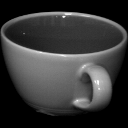

Supplement: Supplemental Information 1 [file peerj-cs-10-1906-s001.zip › coil-20/obj18__13.png]

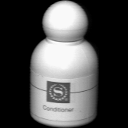

Supplement: Supplemental Information 1 [file peerj-cs-10-1906-s001.zip › coil-20/obj16__1.png]

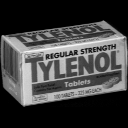

Supplement: Supplemental Information 1 [file peerj-cs-10-1906-s001.zip › coil-20/obj9__67.png]

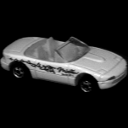

Supplement: Supplemental Information 1 [file peerj-cs-10-1906-s001.zip › coil-20/obj19__41.png]

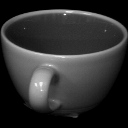

Supplement: Supplemental Information 1 [file peerj-cs-10-1906-s001.zip › coil-20/obj18__20.png]

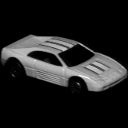

Supplement: Supplemental Information 1 [file peerj-cs-10-1906-s001.zip › coil-20/obj3__43.png]

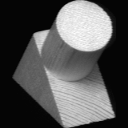

Supplement: Supplemental Information 1 [file peerj-cs-10-1906-s001.zip › coil-20/obj2__49.png]

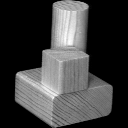

Supplement: Supplemental Information 1 [file peerj-cs-10-1906-s001.zip › coil-20/obj7__11.png]

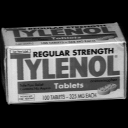

Supplement: Supplemental Information 1 [file peerj-cs-10-1906-s001.zip › coil-20/obj9__70.png]

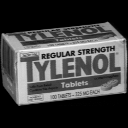

Supplement: Supplemental Information 1 [file peerj-cs-10-1906-s001.zip › coil-20/obj9__68.png]

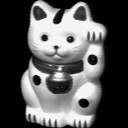

Supplement: Supplemental Information 1 [file peerj-cs-10-1906-s001.zip › coil-20/obj4__1.png]

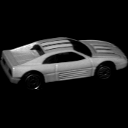

Supplement: Supplemental Information 1 [file peerj-cs-10-1906-s001.zip › coil-20/obj3__32.png]

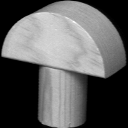

Supplement: Supplemental Information 1 [file peerj-cs-10-1906-s001.zip › coil-20/obj11__5.png]

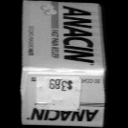

Supplement: Supplemental Information 1 [file peerj-cs-10-1906-s001.zip › coil-20/obj5__17.png]

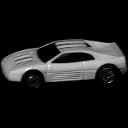

Supplement: Supplemental Information 1 [file peerj-cs-10-1906-s001.zip › coil-20/obj3__69.png]

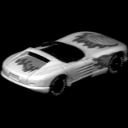

Supplement: Supplemental Information 1 [file peerj-cs-10-1906-s001.zip › coil-20/obj6__27.png]

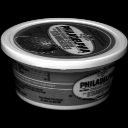

Supplement: Supplemental Information 1 [file peerj-cs-10-1906-s001.zip › coil-20/obj20__14.png]

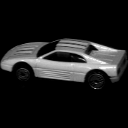

Supplement: Supplemental Information 1 [file peerj-cs-10-1906-s001.zip › coil-20/obj3__2.png]

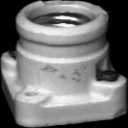

Supplement: Supplemental Information 1 [file peerj-cs-10-1906-s001.zip › coil-20/obj14__33.png]

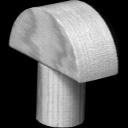

Supplement: Supplemental Information 1 [file peerj-cs-10-1906-s001.zip › coil-20/obj11__10.png]

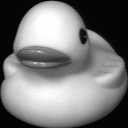

Supplement: Supplemental Information 1 [file peerj-cs-10-1906-s001.zip › coil-20/obj1__57.png]

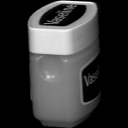

Supplement: Supplemental Information 1 [file peerj-cs-10-1906-s001.zip › coil-20/obj10__57.png]

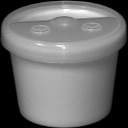

Supplement: Supplemental Information 1 [file peerj-cs-10-1906-s001.zip › coil-20/obj15__34.png]

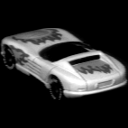

Supplement: Supplemental Information 1 [file peerj-cs-10-1906-s001.zip › coil-20/obj6__11.png]

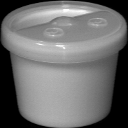

Supplement: Supplemental Information 1 [file peerj-cs-10-1906-s001.zip › coil-20/obj15__29.png]

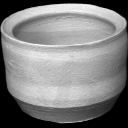

Supplement: Supplemental Information 1 [file peerj-cs-10-1906-s001.zip › coil-20/obj17__40.png]

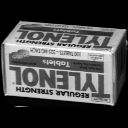

Supplement: Supplemental Information 1 [file peerj-cs-10-1906-s001.zip › coil-20/obj9__38.png]

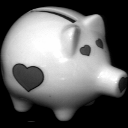

Supplement: Supplemental Information 1 [file peerj-cs-10-1906-s001.zip › coil-20/obj13__42.png]

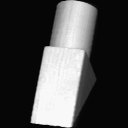

Supplement: Supplemental Information 1 [file peerj-cs-10-1906-s001.zip › coil-20/obj2__20.png]

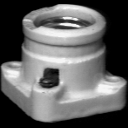

Supplement: Supplemental Information 1 [file peerj-cs-10-1906-s001.zip › coil-20/obj14__12.png]

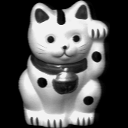

Supplement: Supplemental Information 1 [file peerj-cs-10-1906-s001.zip › coil-20/obj4__70.png]

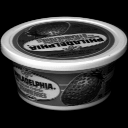

Supplement: Supplemental Information 1 [file peerj-cs-10-1906-s001.zip › coil-20/obj20__32.png]

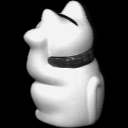

Supplement: Supplemental Information 1 [file peerj-cs-10-1906-s001.zip › coil-20/obj4__26.png]

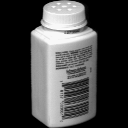

Supplement: Supplemental Information 1 [file peerj-cs-10-1906-s001.zip › coil-20/obj8__29.png]

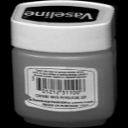

Supplement: Supplemental Information 1 [file peerj-cs-10-1906-s001.zip › coil-20/obj10__37.png]

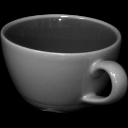

Supplement: Supplemental Information 1 [file peerj-cs-10-1906-s001.zip › coil-20/obj18__10.png]

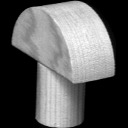

Supplement: Supplemental Information 1 [file peerj-cs-10-1906-s001.zip › coil-20/obj11__11.png]

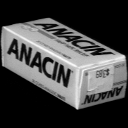

Supplement: Supplemental Information 1 [file peerj-cs-10-1906-s001.zip › coil-20/obj5__10.png]

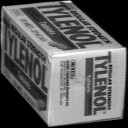

Supplement: Supplemental Information 1 [file peerj-cs-10-1906-s001.zip › coil-20/obj9__13.png]

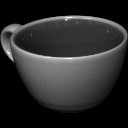

Supplement: Supplemental Information 1 [file peerj-cs-10-1906-s001.zip › coil-20/obj18__44.png]

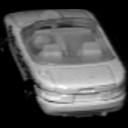

Supplement: Supplemental Information 1 [file peerj-cs-10-1906-s001.zip › coil-20/obj19__15.png]

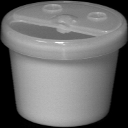

Supplement: Supplemental Information 1 [file peerj-cs-10-1906-s001.zip › coil-20/obj15__5.png]

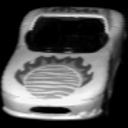

Supplement: Supplemental Information 1 [file peerj-cs-10-1906-s001.zip › coil-20/obj6__55.png]

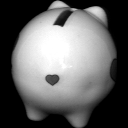

Supplement: Supplemental Information 1 [file peerj-cs-10-1906-s001.zip › coil-20/obj13__21.png]

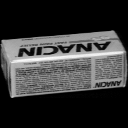

Supplement: Supplemental Information 1 [file peerj-cs-10-1906-s001.zip › coil-20/obj5__33.png]

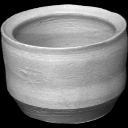

Supplement: Supplemental Information 1 [file peerj-cs-10-1906-s001.zip › coil-20/obj17__36.png]

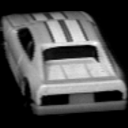

Supplement: Supplemental Information 1 [file peerj-cs-10-1906-s001.zip › coil-20/obj3__15.png]

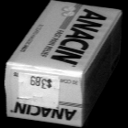

Supplement: Supplemental Information 1 [file peerj-cs-10-1906-s001.zip › coil-20/obj5__20.png]

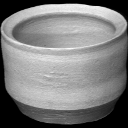

Supplement: Supplemental Information 1 [file peerj-cs-10-1906-s001.zip › coil-20/obj17__31.png]

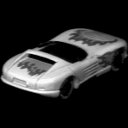

Supplement: Supplemental Information 1 [file peerj-cs-10-1906-s001.zip › coil-20/obj6__25.png]

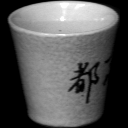

Supplement: Supplemental Information 1 [file peerj-cs-10-1906-s001.zip › coil-20/obj12__59.png]

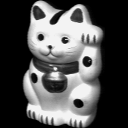

Supplement: Supplemental Information 1 [file peerj-cs-10-1906-s001.zip › coil-20/obj4__3.png]

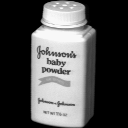

Supplement: Supplemental Information 1 [file peerj-cs-10-1906-s001.zip › coil-20/obj8__3.png]
