# Supplementary figures and images for: AMCFCN: attentive multi-view contrastive fusion clustering net (part 1 of 3)
Source: PeerJ Comput Sci. 2024 Mar 5;10:e1906. doi: 10.7717/peerj-cs.1906 (PMC11636696; doi:10.7717/peerj-cs.1906)

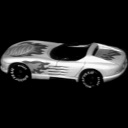

Supplement: Supplemental Information 1 [file peerj-cs-10-1906-s001.zip › coil-20/obj6__2.png]

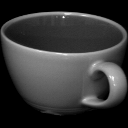

Supplement: Supplemental Information 1 [file peerj-cs-10-1906-s001.zip › coil-20/obj18__11.png]

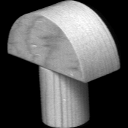

Supplement: Supplemental Information 1 [file peerj-cs-10-1906-s001.zip › coil-20/obj11__45.png]

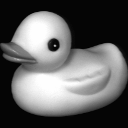

Supplement: Supplemental Information 1 [file peerj-cs-10-1906-s001.zip › coil-20/obj1__65.png]

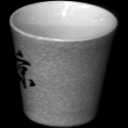

Supplement: Supplemental Information 1 [file peerj-cs-10-1906-s001.zip › coil-20/obj12__15.png]

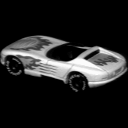

Supplement: Supplemental Information 1 [file peerj-cs-10-1906-s001.zip › coil-20/obj6__6.png]

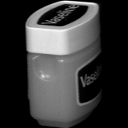

Supplement: Supplemental Information 1 [file peerj-cs-10-1906-s001.zip › coil-20/obj10__58.png]

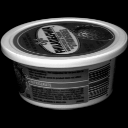

Supplement: Supplemental Information 1 [file peerj-cs-10-1906-s001.zip › coil-20/obj20__51.png]

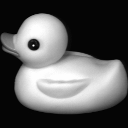

Supplement: Supplemental Information 1 [file peerj-cs-10-1906-s001.zip › coil-20/obj1__1.png]

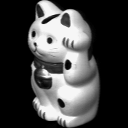

Supplement: Supplemental Information 1 [file peerj-cs-10-1906-s001.zip › coil-20/obj4__9.png]

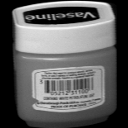

Supplement: Supplemental Information 1 [file peerj-cs-10-1906-s001.zip › coil-20/obj10__34.png]

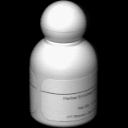

Supplement: Supplemental Information 1 [file peerj-cs-10-1906-s001.zip › coil-20/obj16__26.png]

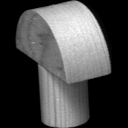

Supplement: Supplemental Information 1 [file peerj-cs-10-1906-s001.zip › coil-20/obj11__48.png]

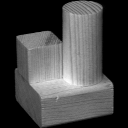

Supplement: Supplemental Information 1 [file peerj-cs-10-1906-s001.zip › coil-20/obj7__31.png]

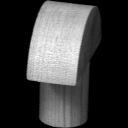

Supplement: Supplemental Information 1 [file peerj-cs-10-1906-s001.zip › coil-20/obj11__20.png]

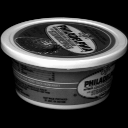

Supplement: Supplemental Information 1 [file peerj-cs-10-1906-s001.zip › coil-20/obj20__13.png]

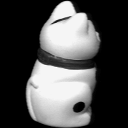

Supplement: Supplemental Information 1 [file peerj-cs-10-1906-s001.zip › coil-20/obj4__49.png]

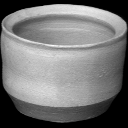

Supplement: Supplemental Information 1 [file peerj-cs-10-1906-s001.zip › coil-20/obj17__4.png]

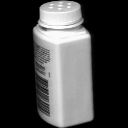

Supplement: Supplemental Information 1 [file peerj-cs-10-1906-s001.zip › coil-20/obj8__48.png]

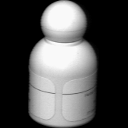

Supplement: Supplemental Information 1 [file peerj-cs-10-1906-s001.zip › coil-20/obj16__18.png]

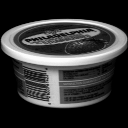

Supplement: Supplemental Information 1 [file peerj-cs-10-1906-s001.zip › coil-20/obj20__66.png]

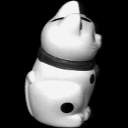

Supplement: Supplemental Information 1 [file peerj-cs-10-1906-s001.zip › coil-20/obj4__53.png]

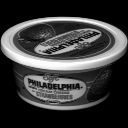

Supplement: Supplemental Information 1 [file peerj-cs-10-1906-s001.zip › coil-20/obj20__26.png]

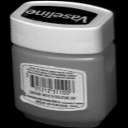

Supplement: Supplemental Information 1 [file peerj-cs-10-1906-s001.zip › coil-20/obj10__40.png]

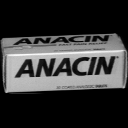

Supplement: Supplemental Information 1 [file peerj-cs-10-1906-s001.zip › coil-20/obj5__70.png]

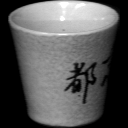

Supplement: Supplemental Information 1 [file peerj-cs-10-1906-s001.zip › coil-20/obj12__60.png]

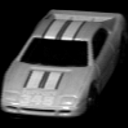

Supplement: Supplemental Information 1 [file peerj-cs-10-1906-s001.zip › coil-20/obj3__56.png]

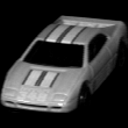

Supplement: Supplemental Information 1 [file peerj-cs-10-1906-s001.zip › coil-20/obj3__57.png]

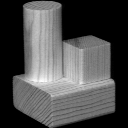

Supplement: Supplemental Information 1 [file peerj-cs-10-1906-s001.zip › coil-20/obj7__65.png]

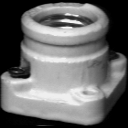

Supplement: Supplemental Information 1 [file peerj-cs-10-1906-s001.zip › coil-20/obj14__21.png]

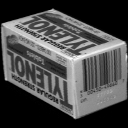

Supplement: Supplemental Information 1 [file peerj-cs-10-1906-s001.zip › coil-20/obj9__46.png]

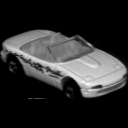

Supplement: Supplemental Information 1 [file peerj-cs-10-1906-s001.zip › coil-20/obj19__45.png]

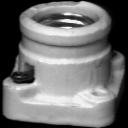

Supplement: Supplemental Information 1 [file peerj-cs-10-1906-s001.zip › coil-20/obj14__19.png]

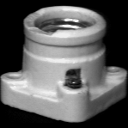

Supplement: Supplemental Information 1 [file peerj-cs-10-1906-s001.zip › coil-20/obj14__6.png]

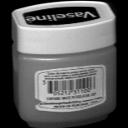

Supplement: Supplemental Information 1 [file peerj-cs-10-1906-s001.zip › coil-20/obj10__33.png]

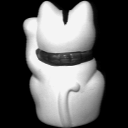

Supplement: Supplemental Information 1 [file peerj-cs-10-1906-s001.zip › coil-20/obj4__35.png]

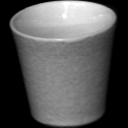

Supplement: Supplemental Information 1 [file peerj-cs-10-1906-s001.zip › coil-20/obj12__44.png]

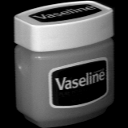

Supplement: Supplemental Information 1 [file peerj-cs-10-1906-s001.zip › coil-20/obj10__65.png]

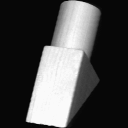

Supplement: Supplemental Information 1 [file peerj-cs-10-1906-s001.zip › coil-20/obj2__21.png]

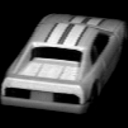

Supplement: Supplemental Information 1 [file peerj-cs-10-1906-s001.zip › coil-20/obj3__20.png]

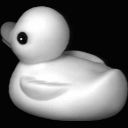

Supplement: Supplemental Information 1 [file peerj-cs-10-1906-s001.zip › coil-20/obj1__6.png]

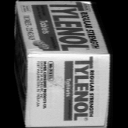

Supplement: Supplemental Information 1 [file peerj-cs-10-1906-s001.zip › coil-20/obj9__16.png]

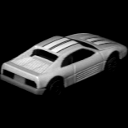

Supplement: Supplemental Information 1 [file peerj-cs-10-1906-s001.zip › coil-20/obj3__26.png]

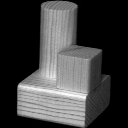

Supplement: Supplemental Information 1 [file peerj-cs-10-1906-s001.zip › coil-20/obj7__4.png]

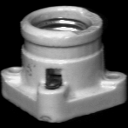

Supplement: Supplemental Information 1 [file peerj-cs-10-1906-s001.zip › coil-20/obj14__47.png]

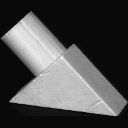

Supplement: Supplemental Information 1 [file peerj-cs-10-1906-s001.zip › coil-20/obj2__3.png]

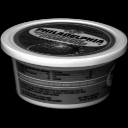

Supplement: Supplemental Information 1 [file peerj-cs-10-1906-s001.zip › coil-20/obj20__5.png]

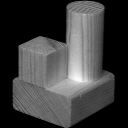

Supplement: Supplemental Information 1 [file peerj-cs-10-1906-s001.zip › coil-20/obj7__25.png]

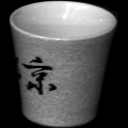

Supplement: Supplemental Information 1 [file peerj-cs-10-1906-s001.zip › coil-20/obj12__9.png]

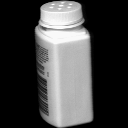

Supplement: Supplemental Information 1 [file peerj-cs-10-1906-s001.zip › coil-20/obj8__49.png]

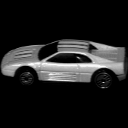

Supplement: Supplemental Information 1 [file peerj-cs-10-1906-s001.zip › coil-20/obj3__0.png]

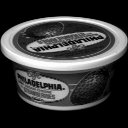

Supplement: Supplemental Information 1 [file peerj-cs-10-1906-s001.zip › coil-20/obj20__30.png]

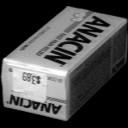

Supplement: Supplemental Information 1 [file peerj-cs-10-1906-s001.zip › coil-20/obj5__22.png]

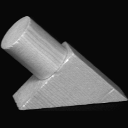

Supplement: Supplemental Information 1 [file peerj-cs-10-1906-s001.zip › coil-20/obj2__69.png]

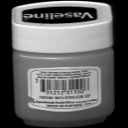

Supplement: Supplemental Information 1 [file peerj-cs-10-1906-s001.zip › coil-20/obj10__35.png]

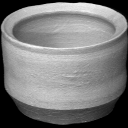

Supplement: Supplemental Information 1 [file peerj-cs-10-1906-s001.zip › coil-20/obj17__27.png]

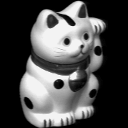

Supplement: Supplemental Information 1 [file peerj-cs-10-1906-s001.zip › coil-20/obj4__64.png]

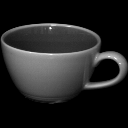

Supplement: Supplemental Information 1 [file peerj-cs-10-1906-s001.zip › coil-20/obj18__4.png]

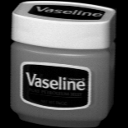

Supplement: Supplemental Information 1 [file peerj-cs-10-1906-s001.zip › coil-20/obj10__2.png]

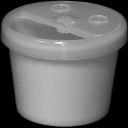

Supplement: Supplemental Information 1 [file peerj-cs-10-1906-s001.zip › coil-20/obj15__8.png]

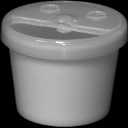

Supplement: Supplemental Information 1 [file peerj-cs-10-1906-s001.zip › coil-20/obj15__4.png]

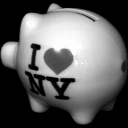

Supplement: Supplemental Information 1 [file peerj-cs-10-1906-s001.zip › coil-20/obj13__6.png]

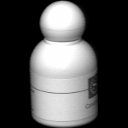

Supplement: Supplemental Information 1 [file peerj-cs-10-1906-s001.zip › coil-20/obj16__58.png]

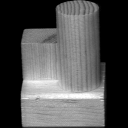

Supplement: Supplemental Information 1 [file peerj-cs-10-1906-s001.zip › coil-20/obj7__36.png]

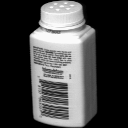

Supplement: Supplemental Information 1 [file peerj-cs-10-1906-s001.zip › coil-20/obj8__40.png]

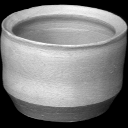

Supplement: Supplemental Information 1 [file peerj-cs-10-1906-s001.zip › coil-20/obj17__1.png]

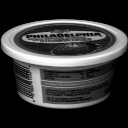

Supplement: Supplemental Information 1 [file peerj-cs-10-1906-s001.zip › coil-20/obj20__70.png]

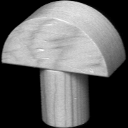

Supplement: Supplemental Information 1 [file peerj-cs-10-1906-s001.zip › coil-20/obj11__40.png]

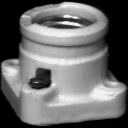

Supplement: Supplemental Information 1 [file peerj-cs-10-1906-s001.zip › coil-20/obj14__14.png]

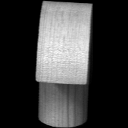

Supplement: Supplemental Information 1 [file peerj-cs-10-1906-s001.zip › coil-20/obj11__18.png]

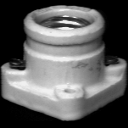

Supplement: Supplemental Information 1 [file peerj-cs-10-1906-s001.zip › coil-20/obj14__25.png]

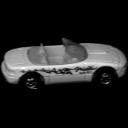

Supplement: Supplemental Information 1 [file peerj-cs-10-1906-s001.zip › coil-20/obj19__34.png]

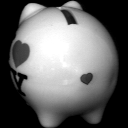

Supplement: Supplemental Information 1 [file peerj-cs-10-1906-s001.zip › coil-20/obj13__14.png]

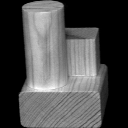

Supplement: Supplemental Information 1 [file peerj-cs-10-1906-s001.zip › coil-20/obj7__55.png]

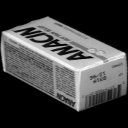

Supplement: Supplemental Information 1 [file peerj-cs-10-1906-s001.zip › coil-20/obj5__46.png]

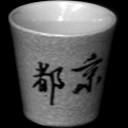

Supplement: Supplemental Information 1 [file peerj-cs-10-1906-s001.zip › coil-20/obj12__68.png]

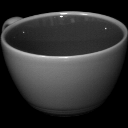

Supplement: Supplemental Information 1 [file peerj-cs-10-1906-s001.zip › coil-20/obj18__46.png]

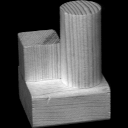

Supplement: Supplemental Information 1 [file peerj-cs-10-1906-s001.zip › coil-20/obj7__33.png]

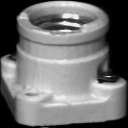

Supplement: Supplemental Information 1 [file peerj-cs-10-1906-s001.zip › coil-20/obj14__69.png]

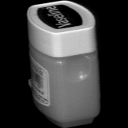

Supplement: Supplemental Information 1 [file peerj-cs-10-1906-s001.zip › coil-20/obj10__50.png]

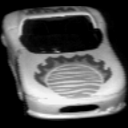

Supplement: Supplemental Information 1 [file peerj-cs-10-1906-s001.zip › coil-20/obj6__52.png]

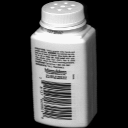

Supplement: Supplemental Information 1 [file peerj-cs-10-1906-s001.zip › coil-20/obj8__39.png]

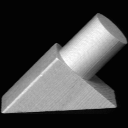

Supplement: Supplemental Information 1 [file peerj-cs-10-1906-s001.zip › coil-20/obj2__35.png]

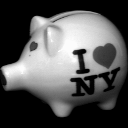

Supplement: Supplemental Information 1 [file peerj-cs-10-1906-s001.zip › coil-20/obj13__67.png]

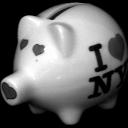

Supplement: Supplemental Information 1 [file peerj-cs-10-1906-s001.zip › coil-20/obj13__62.png]

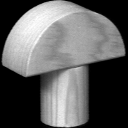

Supplement: Supplemental Information 1 [file peerj-cs-10-1906-s001.zip › coil-20/obj11__65.png]

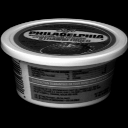

Supplement: Supplemental Information 1 [file peerj-cs-10-1906-s001.zip › coil-20/obj20__1.png]

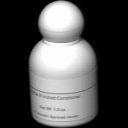

Supplement: Supplemental Information 1 [file peerj-cs-10-1906-s001.zip › coil-20/obj16__40.png]

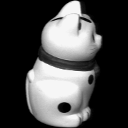

Supplement: Supplemental Information 1 [file peerj-cs-10-1906-s001.zip › coil-20/obj4__54.png]

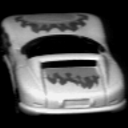

Supplement: Supplemental Information 1 [file peerj-cs-10-1906-s001.zip › coil-20/obj6__16.png]

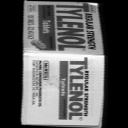

Supplement: Supplemental Information 1 [file peerj-cs-10-1906-s001.zip › coil-20/obj9__17.png]

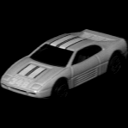

Supplement: Supplemental Information 1 [file peerj-cs-10-1906-s001.zip › coil-20/obj3__61.png]

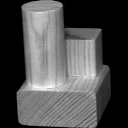

Supplement: Supplemental Information 1 [file peerj-cs-10-1906-s001.zip › coil-20/obj7__56.png]

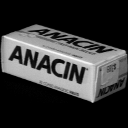

Supplement: Supplemental Information 1 [file peerj-cs-10-1906-s001.zip › coil-20/obj5__7.png]

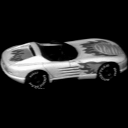

Supplement: Supplemental Information 1 [file peerj-cs-10-1906-s001.zip › coil-20/obj6__33.png]

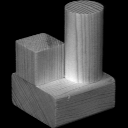

Supplement: Supplemental Information 1 [file peerj-cs-10-1906-s001.zip › coil-20/obj7__28.png]

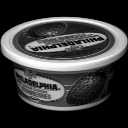

Supplement: Supplemental Information 1 [file peerj-cs-10-1906-s001.zip › coil-20/obj20__31.png]

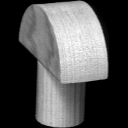

Supplement: Supplemental Information 1 [file peerj-cs-10-1906-s001.zip › coil-20/obj11__13.png]

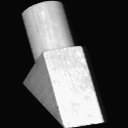

Supplement: Supplemental Information 1 [file peerj-cs-10-1906-s001.zip › coil-20/obj2__13.png]

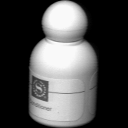

Supplement: Supplemental Information 1 [file peerj-cs-10-1906-s001.zip › coil-20/obj16__8.png]
