# Supplementary figures and images for: AMCFCN: attentive multi-view contrastive fusion clustering net (part 3 of 3)
Source: PeerJ Comput Sci. 2024 Mar 5;10:e1906. doi: 10.7717/peerj-cs.1906 (PMC11636696; doi:10.7717/peerj-cs.1906)

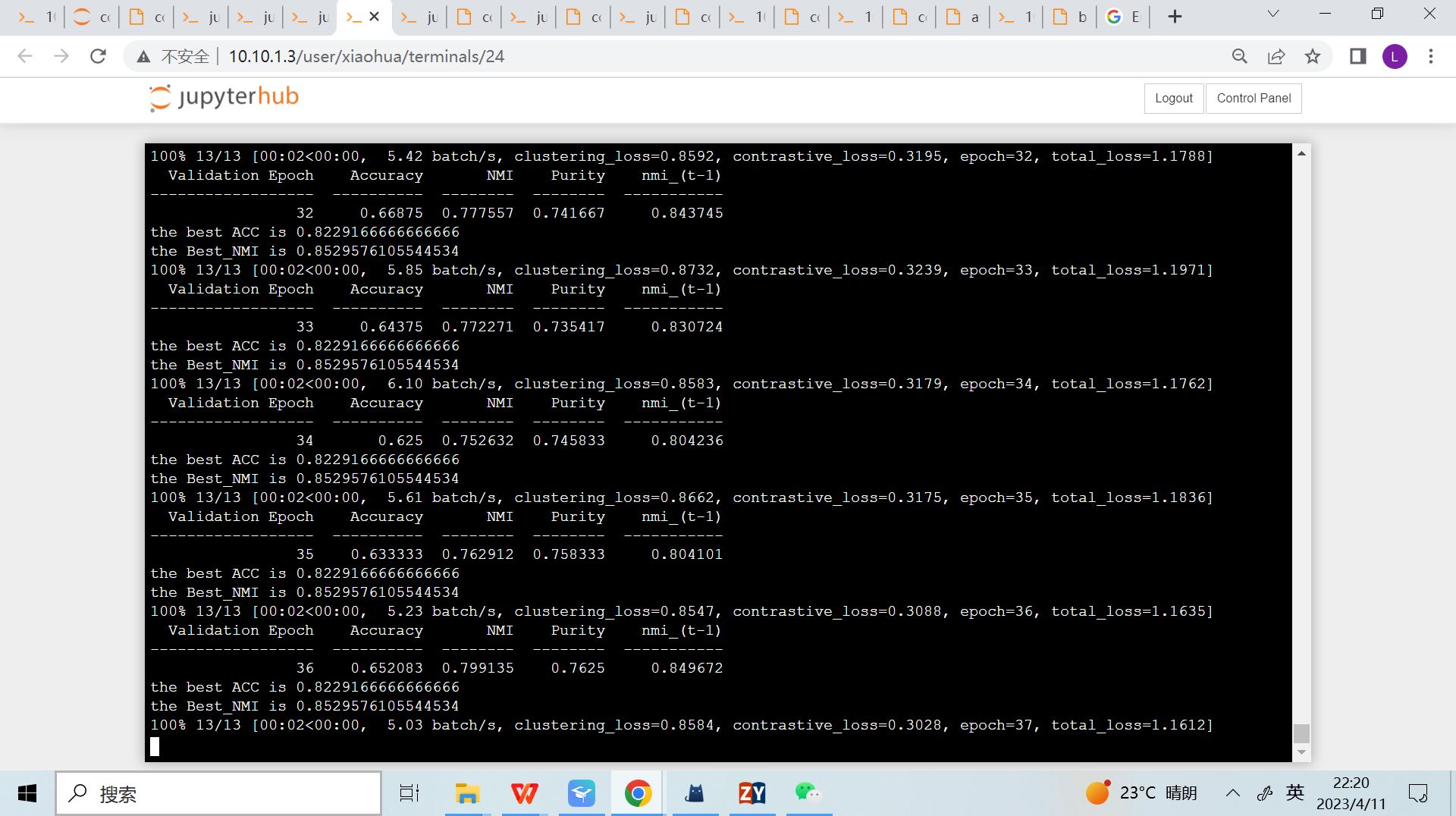

Supplement: Supplemental Information 4 [file peerj-cs-10-1906-s004.jpg]

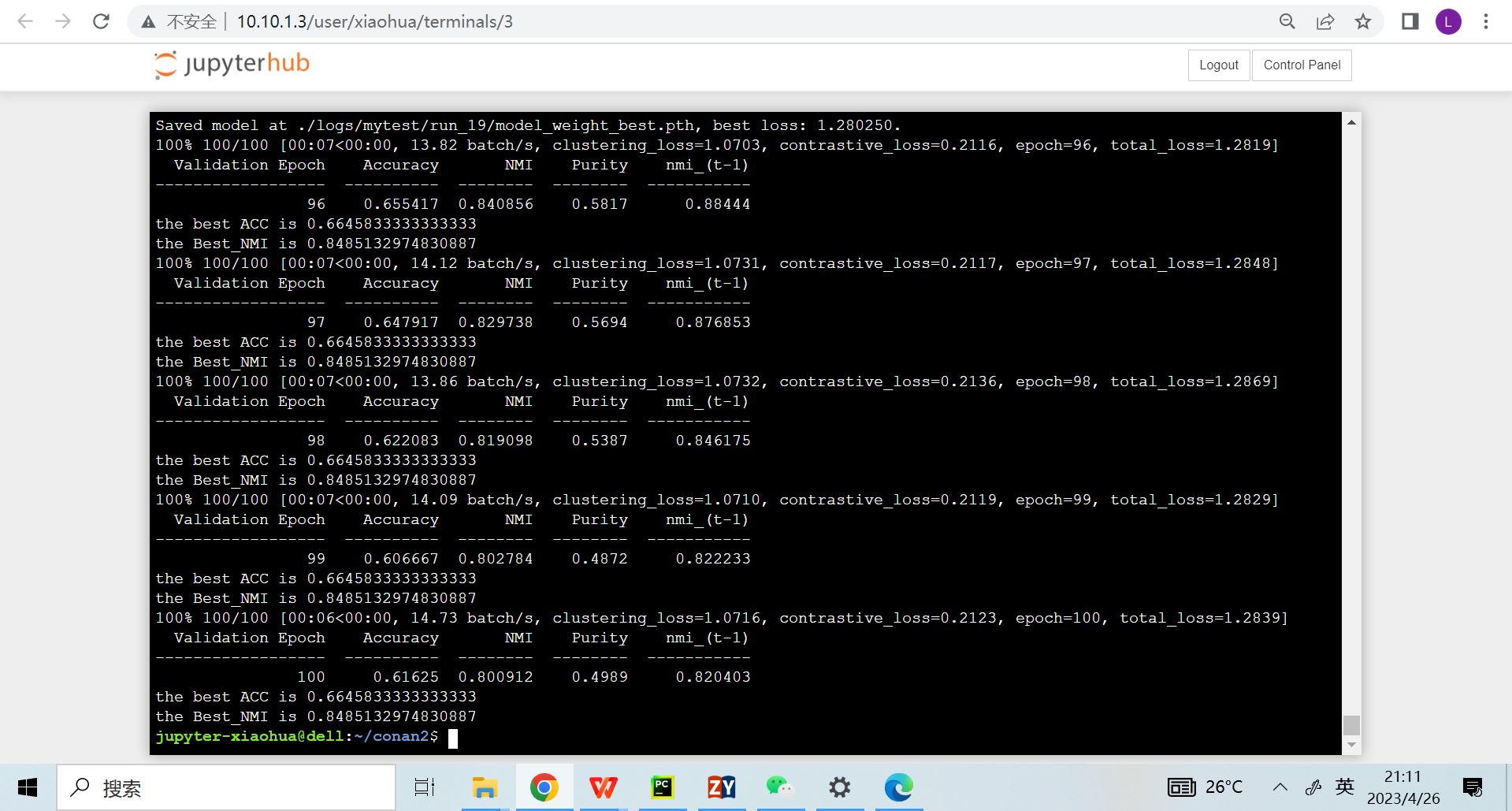

Supplement: Supplemental Information 6 [file peerj-cs-10-1906-s006.jpg]

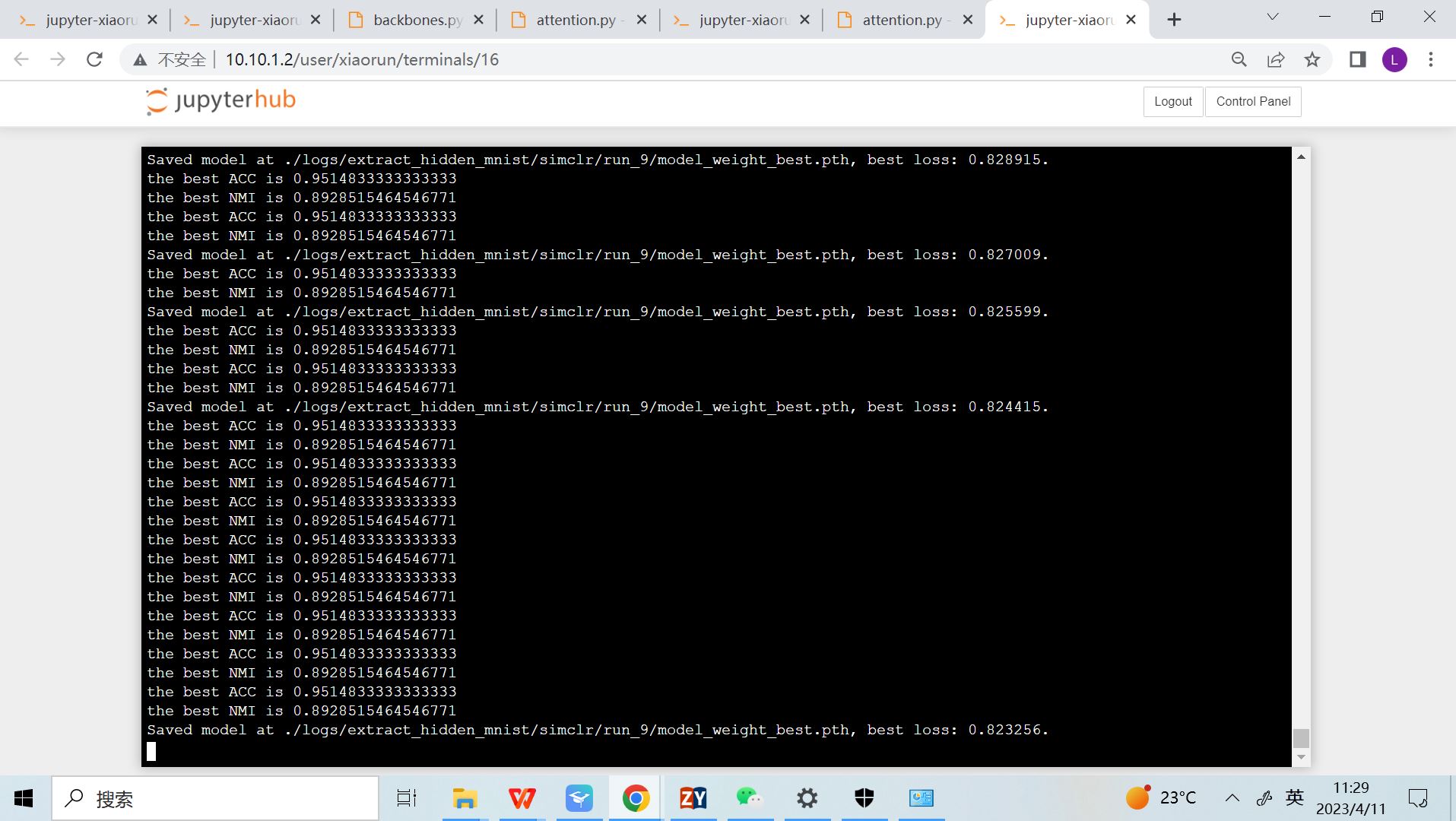

Supplement: Supplemental Information 8 [file peerj-cs-10-1906-s008.jpg]

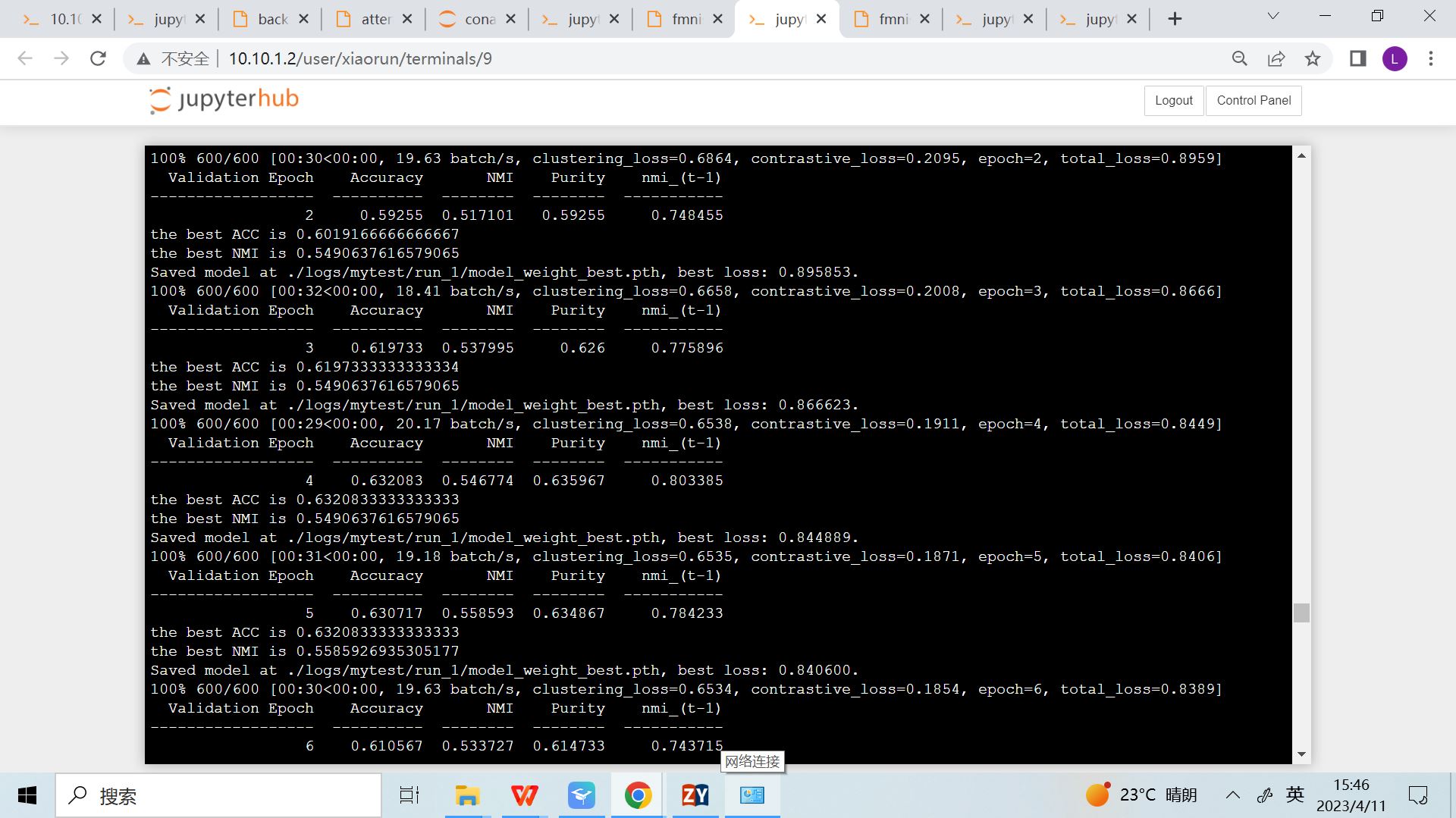

Supplement: Supplemental Information 11 [file peerj-cs-10-1906-s011.jpg]
